# Maternal perspectives on the RSV vaccine (Abrysvo): a thematic analysis of survey findings from the first season of implementation in England and Scotland

**DOI:** 10.1136/archdischild-2025-329427

**Published:** 2026-02-25

**Authors:** Shaun O’Hagan, Thomas C Williams, Robin Marlow, Simon B Drysdale, Steve Cunningham, Helen Elizabeth Groves, Chengetai D Mpamhanga, Mark D Lyttle, Samantha Hunt, Xinxue Liu, Thomas Waterfield, Damian Roland, Dalia Iskander, Kerry Woolfall, on behalf of the PERUKI & BronchStart Collaboration

**Affiliations:** 1 Wellcome Wolfson Institute for Experimental Medicine, Queen’s University Belfast School of Medicine Dentistry and Biomedical Sciences, Belfast, UK; 2 Child Life and Health, University of Edinburgh Division of Clinical and Surgical Sciences, Edinburgh, UK; 3 Department of Paediatric Respiratory and Sleep Medicine, Royal Hospital for Children and Young People, Edinburgh, UK; 4 Population Health Sciences, University of Bristol, Bristol, UK; 5 Emergency Department, Bristol Royal Hospital for Children, Bristol, UK; 6 Oxford Vaccine Group, Department of Paediatrics, University of Oxford, Oxford, UK; 7 NIHR Oxford Biomedical Research Centre, Oxford, UK; 8 The University of Edinburgh Centre for Inflammation Research, Edinburgh, UK; 9 Paediatric Infectious Diseases, Royal Belfast Hospital for Sick Children, Belfast, UK; 10 Research in Emergency Care Avon Collaborative Hub (REACH), University of the West of England, Bristol, UK; 11 Emergency Department, Perth Children's Hospital, Perth, Western Australia, Australia; 12 Paediatric Emergency Medicine Leicester Academic (PEMLA) Group, University Hospitals of Leicester NHS Trust, Leicester, UK; 13 Population Health Sciences, University of Leicester, Leicester, UK; 14 Department of Anthropology, University College London, London, UK; 15 Public Health, Policy and Systems, University of Liverpool, Liverpool, UK

**Keywords:** Child Health, Infectious Disease Medicine, Paediatric Emergency Medicine, Paediatrics, Respiratory Medicine

## Abstract

**Background:**

Respiratory syncytial virus (RSV) is a leading cause of infant hospitalisation due to lower respiratory tract infections. Until 2022, prevention was limited to the costly monoclonal antibody palivizumab. In August 2024, the UK introduced the RSVpreF (Abrysvo, Pfizer) maternal vaccine into its national immunisation schedule. The success of this programme depends not only on vaccine effectiveness, but also on maternal access, acceptance and uptake.

**Objective:**

To explore maternal perspectives on the RSVpreF vaccine and identify barriers and facilitators to vaccine uptake, to inform antenatal education and public health strategies.

**Methods:**

This qualitative analysis is based on free-text survey responses from 388 vaccine-eligible mothers of infants hospitalised with bronchiolitis, lower respiratory tract infection or acute wheeze, collected between September 2024 and March 2025 across 30 sites, as part of the BronchStop study.

**Results:**

Four key themes were identified: (1) access-related barriers to vaccination, (2) insufficient RSV awareness and information to support informed decision-making, (3) vaccine safety concerns and hesitancy and (4) perception of the maternal RSV vaccine as beneficial and protective. These themes were consistent across sociodemographic groups.

**Conclusions:**

Uptake of the maternal vaccine was influenced by barriers to access, informational gaps and perceived safety concerns. Improved vaccine delivery, enhanced awareness and personalised antenatal counselling are essential to increase vaccine uptake. There is an urgent need to address structural inaccessibility and provide tailored antenatal education to address informational gaps. Ongoing qualitative research is crucial to guide targeted public health interventions ahead of future RSV seasons.

## Introduction

 Respiratory syncytial virus (RSV) is the leading cause of lower respiratory tract infection (LRTI) in infants.^1^ Each year, it poses a substantial global disease burden, responsible for over 3 million hospitalisations and 100 000 deaths in children under 5 years of age.^2^ Infants under 6 months old are especially vulnerable, accounting for a third of global hospitalisations and almost half of the RSV-related deaths in this group.^2^

In the UK, RSV-associated respiratory illness in children causes approximately 450 000 general practitioner consultations and 28 500 hospitalisations annually.^3 4^ This carries a considerable economic burden, with an estimated £80 million in annual UK healthcare expenses, caregiver productivity losses and out-of-pocket payments.^5^

Before 2022, the only licensed prophylaxis for RSV infection in infants was palivizumab. However, this monoclonal antibody is costly and requires monthly injections during the RSV season, limiting its use to high-risk infants.^6^ Since 2022, additional passive immunisations against RSV disease in infants have been licenced: a maternal bivalent vaccine, RSVpreF (Abrysvo, Pfizer) and two long-acting monoclonal antibodies, nirsevimab (Beyfortus, Sanofi) and clesrovimab (Enflonsia, MSD).^7 8^ Both nirsevimab and RSVpreF have demonstrated real-world effectiveness against RSV-associated LRTI in infants and offer lower cost and single-dose administration.^9^

Abrysvo regulatory approval was granted following the MATISSE study, which enrolled over 7000 women vaccinated during 24–36 weeks of gestation and demonstrated a vaccine efficacy of 82% at 90 days and 69% at 180 days for the prevention of severe medically-attended RSV infection.^10^ From August 2024, the UK incorporated RSVpreF into its routine immunisation schedule, recommending administration from 28 weeks’ gestation.^11^

The impact of this immunisation programme hinges not only on vaccine effectiveness, but also on accessibility, maternal acceptance and uptake. Introduction of this maternal vaccine comes amid a global decline in childhood vaccinations across both high-income and low-income settings.^12^ Previous qualitative research has shown limited RSV awareness among pregnant women and midwifery staff, highlighting the importance of targeted education to support vaccine implementation.^13^ Similarly, a UK survey of parental views on maternal vaccination and monoclonal antibodies against RSV found limited information and concerns about multiple vaccines during pregnancy contributed to hesitancy, underscoring the need to address informational needs and communicate vaccine safety and efficacy clearly.^14^ However, some researchers have challenged the utility of the term *vaccine hesitancy*, citing its frequent misuse and misclassification.^15^ Professor Heidi Larson, Director of the Vaccine Confidence Project, describes it as ‘a liminal state of uncertainty in making a vaccine decision’^16^; reflecting its broad and dynamic nature, which complicates definition and measurement.

A recent Royal College of Paediatrics and Child Health review described the UK childhood vaccination system as ‘fragmented and hard to navigate’, with research and funding disproportionately focused on vaccine hesitancy, despite access being a major barrier for many willing parents.^17^ Although focused on childhood vaccination, these issues are equally relevant to vaccines in pregnancy. With maternal vaccination rates remaining low in the UK, 54% for Abrysvo in England in February 2025^18^ and 65.6% for pertussis,^19^ improving timely and equitable access to services for all pregnant individuals is an important first step.

### Objective

The aim of this study is to explore and interpret maternal views regarding the RSVpreF vaccine in order to identify perceived barriers and facilitators to vaccine uptake, guide the development of tailored antenatal education and counselling resources and inform ongoing implementation across the UK.

## Methods

### Study design

BronchStop is a multicentre, test-negative case-control study, using the Paediatric Emergency Research in the UK and Ireland network to assess effectiveness of the maternal RSVpreF vaccine in preventing RSV-associated hospitalisation in infants.^20^ Within the BronchStop study, we surveyed vaccine-eligible mothers of infants aged less than 6 months old admitted to hospital with a clinical diagnosis of bronchiolitis, LRTI or first episode of wheeze. Every mother completed the maternal survey as part of their study involvement. From 30 September 2024 to 24 March 2025, survey participants were recruited from 30 National Health Service (NHS) hospital sites across England and Scotland. NHS care is provided free at point of access, both for vaccination and infant care. Informed consent was obtained from all participants, either in person or via telephone.^21 22^ The analysis in this manuscript was prespecified in the study protocol.^16^

### Data collection

The survey questionnaire was designed to explore factors influencing uptake of RSVpreF. The questionnaire was developed in consultation with DI (medical anthropologist) and finalised in alignment with the 5C model of vaccine hesitancy, addressing key psychological antecedents of vaccination: confidence, complacency, constraints, calculation (risk assessment) and collective responsibility.^23^ Participants were asked if they received the RSV vaccine during pregnancy and completed five Likert-scale questions corresponding to each of the 5C constructs. A public and patient involvement group contributed to the survey design, providing feedback to ensure relevance and acceptability to the target population.^20^ Responses ranged from ‘strongly disagree’ to ‘strongly agree’ and were collected using the validated electronic data entry platform, Research Electronic Data Capture.^24^ The survey was administered by healthcare staff in person during the infant’s hospitalisation or via telephone follow-up. A previously published report presents initial results of the Likert-scale data.^25^ The survey also included two free-text questions inviting participants to describe additional factors influencing their vaccine decision-making.^26^ Free-text responses form the basis of the qualitative analysis presented in this manuscript.

### Data analysis

Qualitative analysis of maternal free-text survey responses was conducted using a thematic and broadly interpretive approach. Self-reported maternal ethnicity and Index of Multiple Deprivation (IMD) were also examined in relation to the identified themes. Researchers followed Braun and Clarke’s methodology for thematic analysis, involving: (1) data familiarisation, (2) generating codes, (3) creating themes from codes, (4) reviewing themes, (5) determining theme significance and (6) reporting findings.^27^

Analysis was informed by the authors’ complementary disciplinary perspectives; SOH (Paediatric Infectious Diseases clinician), KW (Professor in Health Research Methodology) and DI (Associate Professor in Medical Anthropology). NVivo software facilitated the coding process.^28^ A codebook was developed (SOH) comprising both *etic* (researcher-derived) and *emic* (participant-derived) codes. To reduce potential bias, the authors met regularly to develop and refine the coding framework collaboratively, adopting a reflexive approach. KW and DI independently reviewed all maternal quotations by theme, with the framework being refined and agreed on by all three authors.

## Results

### Participants

From a total of 733 maternal survey respondents, free-text responses were provided by 388 mothers (53%). Consent rates for study participation varied across study sites, with lowest uptake at St George’s Hospital (24/43, 44% decline rate) and highest uptake at University Hospitals of Leicester NHS Trust (96/99, 3% decline rate). Among mothers included in this analysis, 270/388 (70%) had RSV-positive infants, 90/388 (23%) had RSV-negative infants and RSV status was unknown for 27/388 (7%). The most common maternal age category, estimated by year of birth, was 1990–1995 (111/388, 28.6%). Mothers self-reported their vaccination status and consented to record checks for verification. Regarding RSVpreF vaccination, 77.1% (299/388) were unvaccinated (63.7% confirmed, 13.4% self-reported), 21.2% (82/388) were vaccinated (18.6% confirmed, 2.6% self-reported) and 1.7% (7/388) had an unknown vaccination status. Self-reported maternal ethnicity was predominantly white (321/388, 82.7%), followed by Asian or Asian British (40/388, 10.3%), black, black British, Caribbean or African (9/388, 2.3%), mixed or multiple ethnic groups (5/388, 1.3%) or other (13/388, 3.4%). Based on IMD quintiles, 25.5% (100/388) were from the most deprived quintile (Q1), with the remainder as follows: Q2 (81/388, 20.9%), Q3 (52/388, 13.4%), Q4 (71/388, 18.3%) and Q5 (50/388, 12.9%). IMD data were missing for 9% of mothers (34/388). No differences in themes were observed by socioeconomic status or ethnicity.

### Qualitative analysis

Four principal themes were identified through thematic analysis of maternal survey responses: (1) access-related barriers to vaccination, (2) insufficient RSV awareness and information to support informed decision-making, (3) vaccine safety concerns and hesitancy and (4) perception of the maternal RSV vaccine as beneficial and protective.

#### Access to vaccination

More than half of mothers (210/388, 54%) reported access-related barriers to vaccination.

##### Vaccination not offered or offered too late in pregnancy

Just over a quarter (102/388, 26%) reported that vaccination was not offered or they could not recall it being offered. A third of these mothers indicated they would have accepted vaccination if given the opportunity (table 1). Several mothers expressed disappointment that it was not offered, with one respondent noting: *“[I] would have loved to have the vaccination but it was not at all offered” (participant 185, mixed or multiple ethnic groups, IMD 2*). Others learnt about the vaccine informally through friends and family, suggesting gaps in healthcare communication (table 1). Some mothers (53/388, 14%) were offered vaccination too late in pregnancy or after they had given birth, possibly reflecting challenges during early programme roll-out (table 1).

**Table 1:** Access to vaccination

1.1 Vaccination not offered or offered too late in pregnancy	
Would have accepted vaccination	*“I was never offered it but would have it next time.” Participant 53, white, IMD 1*. *“If I was offered it in pregnancy, I would have deﬁnitely taken it.” Participant 219, white, IMD 1*.
Awareness only through personal networks	*“Not easy to get [the] vaccine. Midwifery team never offered it, heard from a friend, so asked at next appointment.” Participant 354, white, IMD 2*. *“Only heard about the vaccine because [my] sister was offered it, even though [we] were at the same midwife.” Participant 162, white, IMD 3*.
Offered too late in pregnancy	*“[I] was beyond 38 weeks’ gestation at the point of offering vaccination.” Participant 103, white, IMD 4*. *“Offered appointment 2 months after due date.” Participant 184, Asian or British Asian, IMD not provided*.
1.2 Logistical barriers	
Inconvenient clinic locations	*“Vaccine needs to be made more accessible, especially for people who cannot drive. Nearest appointment was about an hour away.” Participant 34, white, IMD not provided*. *“Vaccine very diffcult to get, had to travel far to get, better access would be better.” Participant 35, Asian/British Asian, IMD 4*.
Limited appointment availability	*“Had to request and chase to have vaccine, lack of appointments made it challenging.” Participant 30, white, IMD 1*. *“Asked 3 times to have vaccine in [antenatal] appointments but told would have to go elsewhere and not available at the time.” Participant 184, Asian or British Asian, IMD not provided*. *“No local vaccination sites so went to GP where they were initially reluctant to give it as they don't routinely give to pregnant women; however [I] did end up receiving it there.” Participant 354, white, IMD 2*.
Vaccine shortages and unclear booking processes	*“Low stock at hospital.” Participant 211, white, IMD 2*. *“Got leaﬂet in post but no information on how to book vaccine. Would have liked to have got it, but not easy to make appointment. Midwives kept changing too.” Participant 171, white, IMD 1*.

GP, general practitioner; IMD, Index of Multiple Deprivation.

##### Logistical barriers

Other mothers (47/388, 12%) described logistical hurdles to accessing vaccination, while very few (4/388, 1%) described the vaccine as readily accessible. Challenges included inconvenient clinic locations, lack of integration into routine antenatal visits, limited appointment availability and staff capacity in both general practice and midwifery services, vaccine stock shortages and unclear booking processes (table 1). Time constraints and competing responsibilities, including work and childcare commitments, further hindered access; *“Not given at a routine appointment and [I have] three other children at home who are young”* (participant 167, white, IMD 1).

##### Perceived medical exclusion

A small number of mothers (10/388, 3%) felt medically excluded due to illness or unclear healthcare guidance; *“Was unwell at time of appointment”* (participant 37, white, IMD 3). These missed opportunities highlight the need for clear, condition-specific advice and flexible appointment scheduling to ensure vaccination remains accessible even in the context of maternal health complications.

### RSV awareness and information for decision-making

Nearly a third of mothers (112/388, 29%) reported receiving insufficient information about the maternal vaccine or the risks of RSV in infants, hindering informed decision-making. Lack of awareness of RSV disease severity and the recently introduced maternal vaccine was commonly reported. Perhaps unsurprisingly, healthcare staff played a critical role in shaping maternal vaccine decision-making, with both positive and negative experiences reported. Mothers working in healthcare often drew on professional experiences; *“mum works in healthcare so [has] seen [the] consequences”* (participant 310, white, IMD 3). Several mothers were encouraged by midwives and doctors, valuing and trusting their professional guidance (table 2). Conversely, some mothers encountered negative opinions, were discouraged or felt pressured to accept vaccination by healthcare staff (table 2). Limited proactive communication from healthcare staff was also highlighted, particularly compared with other antenatal vaccines such as pertussis, which were perceived as more embedded in routine care (table 2). Several respondents indicated the vaccine was only briefly mentioned, without further explanation or accessible resources (table 2). Media narratives—both positive and negative—also shaped attitudes and awareness, including radio, news outlets, national headlines and social media. While some valued media coverage, a larger group highlighted a lack of adequate public messaging and suggested more consistent and sustained media campaigns could improve uptake. Overall, mothers emphasised the need for clear, consistent and easily accessible information to support informed decision-making.

**Table 2:** RSV awareness and information for decision-making

Limited awareness and information	*“Mum was offered but wasn’t given enough information about the vaccine which was newly released, more information should be given. She was initially confused and the importance of getting it wasn’t highlighted to her.” Participant 411, black, black British, Caribbean or African, IMD 1*.
Positive influence of trusted healthcare professionals	*“Through information from [the] midwife [I] wanted to get the vaccine” Participant 346, white, IMD 2*. *“If it’s put forward by the medical staff, I believe in it.” Participant 365, maternal ethnicity not provided, IMD 3*.
Concern due to healthcare staff communication	*“Discussed with midwife and described being ‘put off’ having it as it was a new vaccine.” Participant 359, white, IMD 4*. *“Midwife did not seem confident in the vaccine.” Participant 387, Asian or Asian British, IMD 2*.
Felt pressured by healthcare staff	*“Mum felt that midwife was quite ﬁrm on the benefits of having the vaccine. Mum now feels there is no benefit as baby still caught RSV and ended up in hospital.” Participant 428, white, IMD 4*.
Embedded education	*“Lack of information regarding the vaccine, would like to see more education on RSV, embedded process during pregnancy as lots on whooping cough vaccine but none on RSV.” Participant 17, white, IMD 5*.
Lack of accessible resources	*“I would like more information so people know about it, where to get it, side effects and things that will help to be more informed during the antenatal care, leaflets etc.” Participant 340, Arab, IMD not provided*.
Social media	*“Saw negative posts on social media advising against the vaccine in America.” Participant 139, white, IMD 1*.

IMD, Index of Multiple Deprivation; RSV, respiratory syncytial virus.

### Vaccine safety concerns and hesitancy

Concerns about vaccine safety and its recent introduction were reported by approximately 10% of mothers (38/388). Uncertainty about long-term safety and insufficient information contributed to distrust of the ‘new’ vaccine. This caused one participant to describe feeling like a ‘*guinea pig’* (participant 275, Asian or Asian British, IMD 5). Several mothers declined the vaccine because they had not received it in previous pregnancies and felt reassured by these prior experiences. Others believed communication emphasising the vaccine was not entirely ‘new’, but recently introduced into routine practice, could improve acceptance (table 3). Some mothers indicated they would consider vaccination in the future, once more safety data become available (table 3). Safety concerns were also influenced by prior adverse vaccine experiences, fear of side-effects, apprehension about vaccine-induced illness, perceived risks to their baby, including premature delivery and misconceptions that the vaccine was still in clinical trials (table 3). A very small number of mothers (4/388, 1%) expressed broader vaccine hesitancy related to partner influence, belief in natural immunity or needle phobia (table 3). In some cases, mistrust extended to communication methods, with one mother describing the appointment reminder *“text [as] look[ing] like a scam”* (participant 17, white, IMD 5). These varied concerns underscore the importance of clear, evidence-based communication addressing vaccine safety, side-effects and pregnancy-specific concerns.

**Table 3:** Vaccine safety concerns and hesitancy

Communication	*“Uptake may be higher if clearer that it is not a new vaccine just a new vaccine being offered routinely on [the] NHS.” Participant 174, white, IMD 5*.
Safety data	*“Too early on in the roll-out of the vaccine, would have had it if it had been available for a few years and known it was safe.” Participant 120, white, IMD not provided*.
Adverse reactions	*“After having reactions to covid jabs decided not to have it.” Participant 279, white, IMD 4*.
Concern about risk to the baby	*“Was concerned about risk of prematurity.” Participant 204, white, IMD 2*.
Vaccine hesitancy	*“Decision was due to partners’ hesitancy in vaccine safety.” Participant 6, white, IMD 5*.

IMD, Index of Multiple Deprivation; NHS, National Health Service; RSV, respiratory syncytial virus.

### Vaccine perceived as beneficial and protective

The maternal vaccine was expressed by a number of mothers (52/388, 13%) as an important way to protect their baby. Protective motivation was particularly evident among those with personal or professional experiences of RSV or serious respiratory illness (table 4). Discussions with friends and family, awareness of RSV risks, prior RSV-related hospitalisation in siblings or relatives, winter births and exposure through older siblings were key motivators for mothers to seek vaccination (table 4). For a small proportion (9/388, 2%), RSV seasonality shaped risk perception, with winter pregnancies viewed as making vaccination particularly important (table 4).

**Table 4:** Vaccine perceived as beneficial and protective

Protective motivation	*“Had another premature baby who had RSV, was keen to get vaccine to try and avoid a repeat.” Participant 37, white, IMD 3*.
Previous experiences	*“My baby has siblings and they could bring home stuff from school. My niece had RSV when she was 1 month old and so I was aware how bad it could be.” Participant 393, white, IMD 4*.
RSV seasonality	*“I took it because I was having a winter baby, probably wouldn’t have if it had been summer.” Participant 238, white, IMD 1*.

IMD, Index of Multiple Deprivation.

## Discussion

This study provides valuable insights into barriers and facilitators influencing uptake of the maternal RSVpreF vaccine in the UK. The central identified theme was systemic barriers to vaccination, with many mothers reporting the RSV vaccine was not offered, offered too late in pregnancy or was inaccessible. This is a major obstacle to realising the protective potential of maternal RSV vaccination. Improving uptake requires streamlined vaccine delivery, alongside enhanced healthcare communication and education. Additionally, the seasonality of RSV presents an opportunity to align future public health campaigns with periods of heightened risk, when perceived need and benefit may be greatest. While vaccine hesitancy is often cited as a key barrier, our findings indicate many pregnant individuals are open to vaccination, but hindered by a fragmented and difficult-to-access system.

These findings reflect existing evidence linking low awareness of RSV with reduced acceptance of maternal and infant immunisation.^14 29^ Healthcare provider recommendation, perceived risks and benefits, sociodemographic background and access to care have all been shown to influence antenatal vaccine uptake.^30^ Our study reinforces these findings, particularly the importance of healthcare access. Notably, several mothers spontaneously called for public health campaigns, highlighting both unmet communication needs and readiness to engage when information is accessible.

Many respondents reported inadequate information to make informed decisions, particularly regarding vaccine efficacy, safety and RSV disease severity. Mothers expressed a desire for clear, evidence-based information and balanced antenatal discussions delivered by trusted healthcare professionals, supported by credible published evidence, over passive communication, such as posters, automated texts or information leaflets. Healthcare professionals should also address common misconceptions, including concerns the vaccine is too new, still in trial or increases RSV risk in infants. Tailored education, integrated into routine antenatal care, may further improve vaccine confidence and acceptance. Addressing negative perceptions among healthcare professionals is also essential, as these can undermine maternal confidence and contribute to low antenatal vaccine uptake.

Recent pertussis outbreaks have considerably raised public awareness and concern, contributing to an increase in maternal pertussis vaccination rates: 72% in September 2025, compared with 58% in 2023, according to UKHSA data.^31^ While RSVpreF uptake remains lower than established maternal vaccines, like Tdap, this disparity is likely multifactorial. Early implementation challenges, limited professional and public awareness and safety concerns about a new vaccine likely contributed to these differences. Our qualitative analysis emphasises the need to address awareness and access barriers as the RSVpreF campaign matures. Real-world data from Spain demonstrates nirsevimab uptake among eligible infants reached up to 90% during the first season of implementation,^32^ possibly reflecting easier access and higher acceptability of interventions delivered to newborns. Supporting this, a Scottish cohort study using uptake of intramuscular vitamin K at birth as a proxy for nirsevimab acceptance reported 95.5% coverage between 2019 and 2021.^33^ At present, RSVpreF vaccination in the UK is unlikely to achieve similarly high coverage to produce a comparable population-level impact to that seen in Spain.

### Strengths and limitations

A notable strength of this study is its wide geographical coverage, with responses collected from 30 sites across England and Scotland. This broad reach supports the diversity and representativeness of this sample, including a relatively high proportion self-identifying as not being of white ethnicity (17.6%). This broadly reflects the 2021 Census, which reported 19% of people in England identified as belonging to a non-white ethnic minority group,^34^ but is lower than more recent estimates indicating 28% of mothers in England identified as non-white.^35^

However, there was considerable geographical variability in maternal consent rates, which may have introduced selection bias and affected generalisability. As recruitment was limited to mothers of infants hospitalised with respiratory illness, our findings should also be interpreted in the context of this selected population. These mothers may differ from the wider population in their experiences of infant respiratory disease and perceptions of disease severity, which may limit the generalisability of our results and could plausibly overestimate awareness of, or receptiveness towards, maternal RSVpreF vaccination. However, only 21.2% of participants in our study reported receiving the RSVpreF vaccine, which is lower than early national maternal RSVpreF vaccine coverage reported during programme implementation in England (33.6% in September 2024).^36^ Furthermore, despite any potential overestimation within this population, lack of awareness of both RSV disease in infants and the recently introduced maternal vaccine was a primary theme in our qualitative analysis, underscoring persistent gaps in knowledge required for informed vaccine decision-making.

Just over half of the survey respondents completed the free-text questions, which may have introduced bias, as those holding strong views either for or against vaccination may be over-represented and not representative of the entire study cohort. Maternal awareness of infant RSV status at the time of survey completion likely varied due to differences in testing pathways and survey timing, limiting our ability to assess how RSV status may have influenced survey responses. Surveys were also conducted by healthcare staff, which may have influenced how openly mothers expressed their views. Additionally, this analysis reflects maternal perceptions during the early stages of RSVpreF roll-out in the UK. To address this limitation, the survey will be repeated during the 2025–2026 RSV season, enabling comparative analysis and exploration of evolving attitudes as the programme becomes more established. In parallel, we will conduct a qualitative interview study, *Understanding Parental Decision-Making in RSV Prevention: The BronchStop Qualitative Study,* recruiting through BronchStop and community channels to ensure diverse representation. This study has received ethics approval and will shortly commence purposive recruitment.

## Conclusion

Enhancing maternal RSV vaccine uptake requires a dual focus: addressing systemic barriers to access and integrating RSV education into routine antenatal care. Additionally, providing clear, evidence-based information through trusted healthcare professionals will support informed decision-making and ensure all mothers have the opportunity to benefit from this important public health preventative.

## Data Availability

Data are available upon reasonable request.
